# A simulation approach to assessing vegetation configuration effects on thermal comfort in cold region pocket parks

**DOI:** 10.1038/s41598-025-14749-8

**Published:** 2025-08-06

**Authors:** Qing Wang, Shoucheng Liu, Jingtong Qian

**Affiliations:** College of Forestry and Grassland Science, Jilin Agricultural University, Changchun, 130000 China

**Keywords:** Thermal comfort, Pocket park, Green patterns, Tree coverage, Leaf area density, ENVI-met simulation, Climate-change adaptation, Climate-change mitigation

## Abstract

Pocket parks, play a crucial role in enhancing residents’ thermal comfort and promoting ecological sustainability. Despite their significance, thermal comfort in cold-region spaces remains underexplored, especially vegetation impacts. This study investigates the impact of vegetation configuration on cold-region pocket park thermal environments, using Changchun as a case study via field measurements and ENVI-met simulations. Through the integration of Mean Thermal Sensation Vote (MTSV) and Physiological Equivalent Temperature (PET) indices, the study established the thermal comfort range for Changchun during transitional seasons as 16.69–23.63 ℃, with a thermal neutral temperature of 20.16 ℃. The study developed 27 experimental scenarios to analyze vegetation parameters—tree coverage, Leaf Area Density (LAD), and green patterns. Orthogonal design analysis of simulation results identified the hierarchical impact on thermal comfort: tree coverage > LAD > green patterns. The study proposes three optimal vegetation design strategies for cold-region pocket parks: (1) maintain tree coverage above 50% (ideally 70%); (2) select tree species to complement coverage with LAD of 1.2–1.4; (3) adopt tree-grass combined green patterns for green space layouts. This research presents novel insights and practical guidelines for designing vegetation in micro-scale green spaces in cold-region cities, advocating a sustainable model integrating ecological and social well-being.

## Introduction

Global urbanization is accelerating, with United Nations projections indicating 68% of the global population will reside in cities by 2050^1^, a trend closely mirrored by China’s 65.22% urbanization rate in 2022^2^. The rapid urbanization and large population concentration that have accompanied this process have given rise to a number of urban thermal environmental problems, which have a particularly harsh impact on cities in cold regions ecology. The regulation of the thermal comfort of urban small-scale green spaces represents an important aspect of the improvement of the urban thermal environment^3,4^. As a nature-based solution, plants play a vital role in microclimate regulation^5^. Understanding the mechanisms underlying the thermal comfort effects of different vegetation configuration is crucial for guiding sustainable urban development and fostering resilient urban climates.

Thermal comfort, defined as the subjective satisfaction with thermal environments through psychological perception^6^, is critically influenced by vegetation-mediated microclimate regulation. Studies demonstrate that plant shading (≥ 70% coverage reduces Ta by 2.5–4.5 °C^7)^ and transpiration (urban trees require Leaf Area Index (LAI) ≥ 2.5 for effective thermal comfort improvement^8^) significantly regulate microclimate, while optimized leaf area density (LAD) distribution (e.g., dense-sparse vertical structure) balances shade and ventilation^9,10^. Subsequent studies have refined this understanding by quantifying vegetation parameters: green patterns affect thermal mitigation efficiency in residential areas^11^, tree species selection determines cooling magnitude^12^, canopy height has an effect on Va^13^, while tree coverage correlates with temperature reduction^14^. Recent advances highlight the critical roles of specific metrics—tree coverage modulates G interception^15^, LAD governs microclimate heterogeneity^16^, and optimized configurations yield measurable Physiological Equivalent Temperature (PET) reductions^17^. Research on acoustic-thermal interactions demonstrate the influence of plants on multisensory perception^18^. This evolving knowledge base necessitates systematic investigation into the hierarchical effects of vegetation parameters (green patterns, tree coverage, LAD) for cold-region microclimate design.

Thermal comfort research in urban pocket parks—small-scale green spaces typically ranging from 400 to 10,000 m^2^ with recreational functions^19,20^—has progressed through methodological innovations. Early studies established vegetation’s microclimate regulation via shading and evapotranspiration^9^, while subsequent work quantified species-specific impacts^12^ and spatial configuration effects^11^. Recent advancements integrate ENVI-met simulations to model plant–air interactions^21^, revealing measurable PET reductions through optimized tree coverage^17^ and LAD adjustments^16^. Field-experimental hybrids have further decoded microclimate-thermal perception relationships using wearable sensors^22^ and multivariate regression^23^. Despite these strides, systematic analysis of vegetation parameters in cold-region pocket parks remains underdeveloped, particularly regarding their hierarchical impacts on transitional season comfort—a critical gap this study addresses.

Current understanding of vegetation-mediated thermal comfort in cold-region pocket parks remains nascent, particularly regarding transitional season range. Qixin Liu et al. (2024) dentified a range of UTCI thresholds for cold cities in northern China and did not identify a comfort range for PET^4^. This study employs an integrated approach combining field measurements and ENVI-met simulations to decode hierarchical vegetation impacts (tree coverage, LAD, green patterns). Key innovations include: (1) establishing region-specific comfort benchmarks through psychophysiological index integration; (2) quantifying parameter influence mechanisms via scenario modeling; (3) determining factor hierarchy through orthogonal experimental design; (4) deriving evidence-based design strategies for Changchun’s microclimatic optimization.

## Methodology

### Field survey

#### Study area

**Fig. 1:**
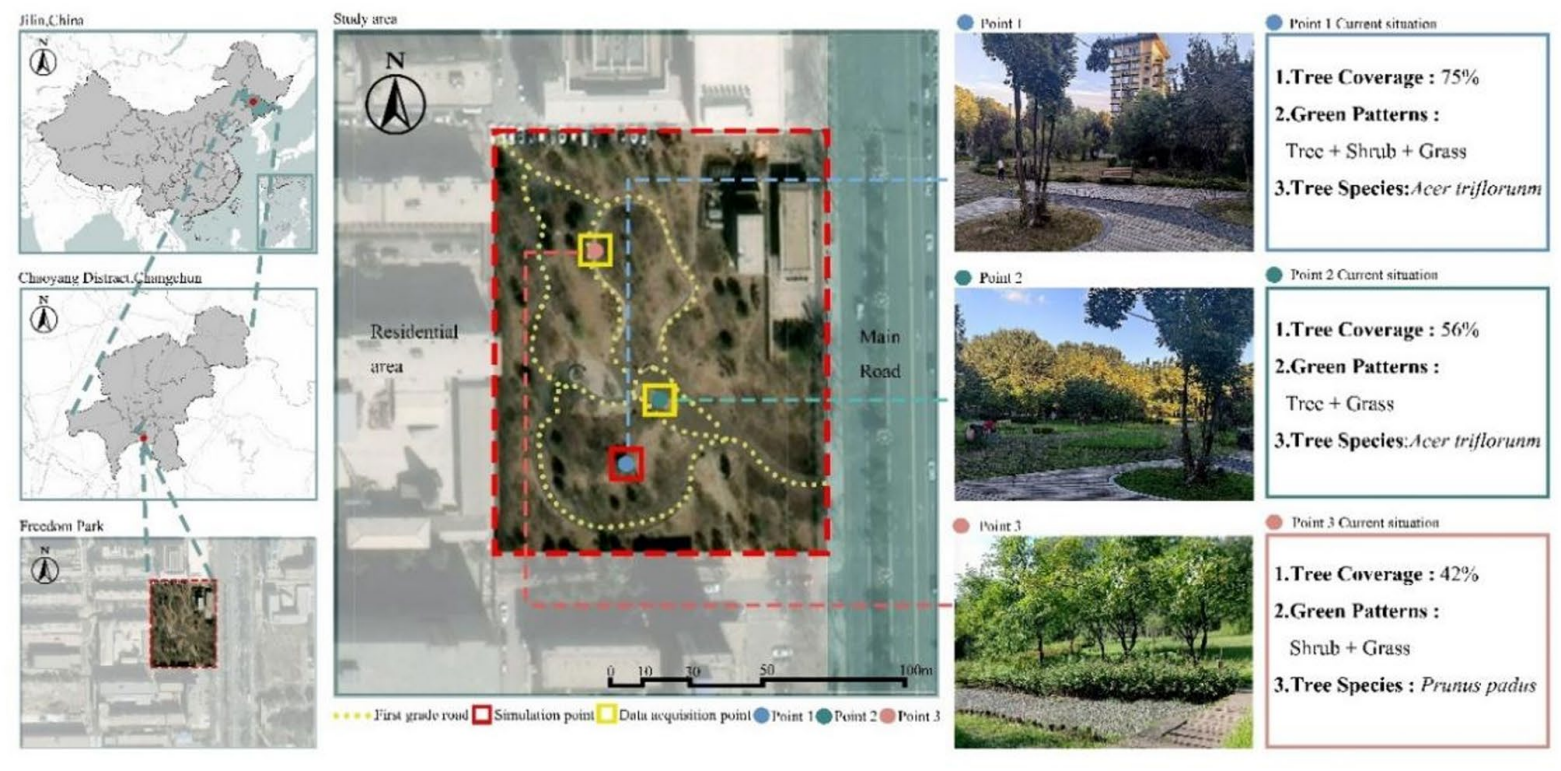
Location of the study area and measuring point selection (Satellite imagery is quoted from tianditu, https://www.tianditu.gov.cn/).

The study area is located in Changchun City, Jilin Province, Northeast China, which has a temperate continental monsoon climate with four distinct seasons. 16 pocket parks were initially screened based on administrative location, primary functions, activity characteristics across functional zones, overall park features, and crowd usage patterns. Through field observations, Freedom Park in Chaoyang District, Changchun City, was ultimately selected as the research subject due to its high pedestrian traffic, vibrant activity levels, and representativeness. Through the field research of Freedom Park, three 10 × 10 m areas^24^ with different green patterns, tree species and tree coverage were selected as the sample squares, among which the sample point 1 with high utilization rate and large flow of people was selected as the subsequent software simulation area, as shown in Fig. 1.

#### Physical parameters measurements

**Fig. 2:**
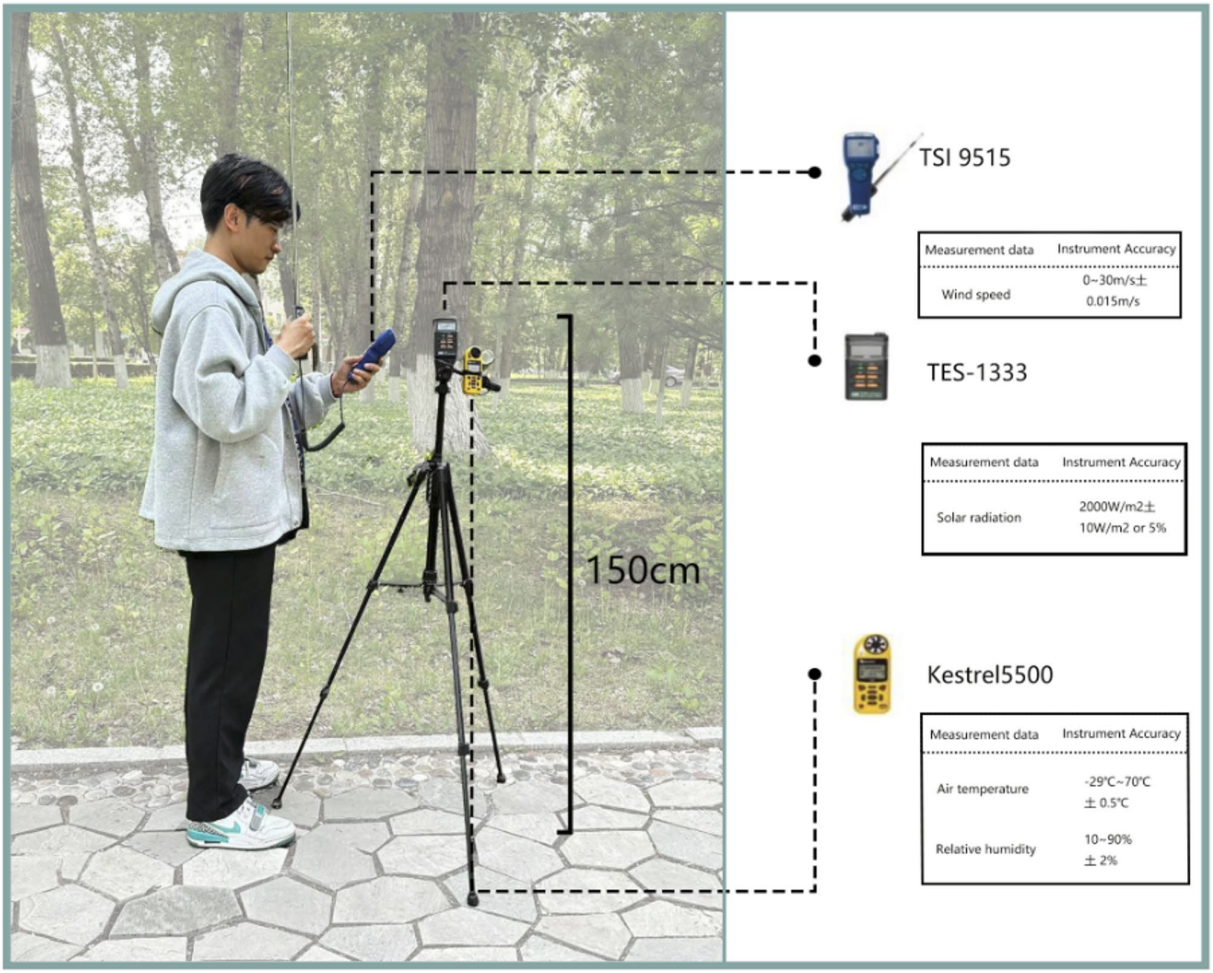
Instruments and measurement methods.

Field measurements were conducted during three characteristic transitional season days (September 15 – 28) under stable meteorological conditions: clear skies, air temperature (Ta) 8 – 22 °C, and scale 1 – 2 winds, with continuous monitoring from 08:00 to 17:00 daily to capture representative data. The equipment used for the experiment and its parameters are referenced in Fig. 2. The Kestrel 5500 was employed to ascertain the Ta, relative humidity (RH). The TSI 9515 hand-held digital wind speed and wind temperature tester was utilized to determine the wind speed (Va), while the TES-1333 was used to measure solar radiation (G). All of the aforementioned test instruments comply with the ISO 7726 standard. The measurement instrument was fixed at about 1.5 m^25^. Meteorological instruments are typically installed between 1.1 and 2 m above ground, with 1.5 m being standard^26^. This height corresponds to the human sensory zone while standing, enabling accurate measurement of microclimate parameters affecting thermal comfort and minimizing ground-level interference, ensuring representative data^27,28^. Data measurements were taken every half an hour, and the average value was taken again at three minutes interval between each measurement period as the final measurement data^29^.

#### Questionnaire survey

The research questionnaire, which consisted of 19 questions divided into three sections, was distributed at each of the three field test area while the actual test was being conducted. (1) basic information survey, including respondents’ age, gender, clothing, and time of arrival, etc.; (2) behavioral situation, including types of behaviors, and time of activities, etc.; (3) evaluation of thermal comfort perceptions, including thermal sensation voting (TSV), thermal comfort voting (TCV) and thermal preference voting (TPV)^30^. TSV is a 5-level evaluation index (− 2 = cold, − 1 = colder, 0 = neutral, 1 = hotter, 2 = hot)^31^. TCV is a 5-level evaluation index (1 = very uncomfortable, 2 = less uncomfortable, 3 = moderate, 4 = more comfortable, 5 = very comfortable)^32^ and TPV is a 5-level evaluation index (− 2 = weaker, − 1 = slightly weaker, 0 = no change, 1 = slightly stronger, 2 = stronger)^33^. By distributing a total of 273 questionnaires for the three different measurement points, invalid questionnaires were excluded and 257 valid questionnaires were obtained after screening, with a pass rate of 94.1%.

#### Selection and calculation of thermal comfort indicators

In this paper, the PET will be calculated using the RayMan Pro Version 3.1 Beta calculation software, the geographical information of the site in terms of latitude and longitude, the current climate data, the personal data, the clothing thermal resistance and the metabolic rate. Based on GB/T 50785 − 2012 standard and combined with the actual situation of clothing during the transition season(long sleeves 0.2clo + jacket 0.35clo + pants 0.25clo + shoes and socks 0.1clo = 0.9clo), this paper took 0.9 clo as the reference value of the clothing thermal resistance in the transition season. The average value of human exercise metabolic rate was calculated by combining the main types of human activity in the free garden obtained from the questionnaire, and it was set to 60 W.

In this study, PET values and mean thermal sensory vote (MTSV) were calculated by linear regression through IBM SPSS Statistics 27 to obtain a regression function fitting equation to determine the PET range scale for pocket parks in Changchun City.

### Vegetation configuration

Since the Albreo plant 3D model construction in the ENVI-met Headquarter 5.6.1 used in the simulation process needs to be achieved by inputting the actual LAD value to achieve the 3D information entry of the plant, this paper uses the LAD index to represent different arbor species.

LAD (leaf area per canopy volume, m^2^/m^3^) quantifies 3D leaf distribution density within the canopy. LAI (leaf area per ground area, m^2^/m^2^) measures total horizontal leaf coverage. LAD better characterizes canopy structure but was unmeasurable due to equipment limits. We derived LAD by inversely calculating region-matched LAI values from literature for simulations.

The common LAI ranges of different plant species in Northeast China were obtained by reviewing the literature^34^, and the LAD ranges were calculated by bringing in the Eqs. (1 and 2) as follows^35^:

1 $$\:\begin{array}{*{20}c} {L\left( z \right) = L_{m} \left( {h - z_{m} /h - z} \right)^{n} exp\left( {\left[ {n\left( {1 - h - z_{m} /h - z } \right)} \right]} \right),n = \left\{ {\begin{array}{*{20}c}{6,0 \le z< z_{m} } \\ {0.5,z_{m} \le z<h} \\ \end{array} } \right.} \\ \end{array}$$

2 $$\:\begin{array}{c}\:LAI={\int\:}_{0}^{n}L\left(z\right)dz\end{array}$$

$$= \int \: _{0}^{h} L_{m} \left( {h - z_{m} /h - z} \right)^{n} exp\left[ {n\left( {1 - h - z_{m} /h - z} \right)} \right]dz$$

Where LAI is the leaf area index, L is the leaf area density (LAD), h is the plant height, and z is the thickness of the stratification.

In this study, the tree cover and vegetation configuration patterns were categorized into three levels (tree coverage: 1 = 30%, 2 = 50%, 3 = 70%; green patterns: 1 = tree, 2 = tree-grass, 3 = tree-shrub-grass) as the quantitative parameters for describing the horizontal and vertical structures, respectively.

### Simulation software selection and calibration

In this paper, we used ENVI-met Headquarter 5.6.1 to simulate the thermal environment of different working conditions in the same square of Freedom Park, and explored the influence mechanism of different plant influences on the microclimate and thermal comfort of urban pocket parks.

According to the site data, the number of modeling grids was 10 m×10 m×15 m, and the unit grid size was 1 m×1 m×1 m. Climatic data conditions were based on the Ta and air humidity of the measured data on site as a criterion. The soil is typical of the Changchun city area, being of the loess-derived chernozem type. And at the same time unified the underlying surfaces and plant spatial layout within the model, the latitude and longitude were inputted according to the actual area of the site, and the rest of the values were defaulted by the software.

In order to further determine the reliability of the simulation software, this paper introduced the root mean square error (RMSE) and mean absolute percentage error (MAPE) to compare and analyze the values of Ta and RH obtained from the simulation at each measurement point with the measured values^36^, and the Eqs. (3 and 4) for each of the two are as follows :

3 $$\:{\text{RMSE}} = \sqrt {\sum \: _{{\text{t}}}^{{\text{n}}} ({\text{A}}_{{\text{t}}} - {\text{F}}_{{\text{t}}} )^{2} /\:{\text{n}}}$$

4 $$\:{\text{MAPE}} = 1/{\text{n}}\sum \: _{{{\text{t}} - 1}}^{{\text{n}}} \left| {{\text{F}}_{{\text{t}}} /{\text{A}}_{{\text{t}}} } \right|$$

At is the simulated value; Ft is the measured value; n is the number of measurements. The calculation results are shown in Table 1.

**Table 1:** Quantitative evaluation between simulated spatial measurement points and measured values.

Meteorological factor	Validation metrics	Point1	Point2	Point3
Air temperature (Ta)	RMSE/℃	2.31	1.38	1.12
Air temperature (Ta)	MAPE/%	9.83	5.16	4.04
Relative humidity (RH)	RMSE/℃	6.73	5.02	1.66
Relative humidity (RH)	MAPE/%	12.66	10.46	4.53

The smaller values of RMSE and MAPE represent the higher accuracy of the model. The model was considered reliable when the RMSE value was less than 1.63 °C, the RH was less than 5%, and the MAPE value Ta and RH were both less than 10%. The simulation software could be used as a numerical simulation tool for the study in this paper.

### Orthogonal test

Orthogonal tests can analyze the various factors that may affect the test results through the available data, and identify the main influencing factors from them. The key to the design of orthogonal test lies in the arrangement of the test factors, which can usually be freely arranged in the columns of the orthogonal table without considering the interaction, as long as no two factors are arranged in a uniform column. A, B, C represent three factors, and 1, 2, and 3 represent three levels of different factors^37^. Taking the outdoor thermal environment evaluation index PET as the test index, the three factors of tree coverage (A), tree species (B) and green patterns (C) were considered, and the orthogonal test factor level table was established by combining the actual factor matching and the simulated environment to further investigate the effect of plant matching form on the thermal environment of the urban pocket park.

## Results

### Measurement data analysis

#### Microclimate data analysis

**Fig. 3:**
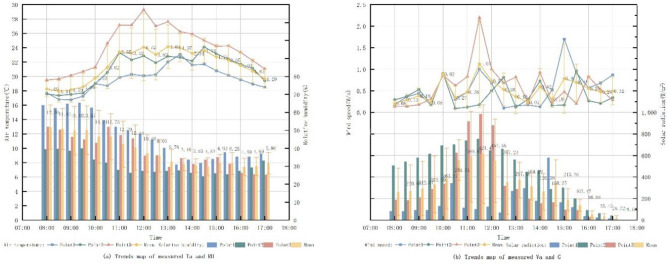
Trend map of measured data on microclimate factors.

Figure 3 presents the microclimatic data recorded at three measurement points within Freedom Park between 08:00 and 17:00 on September 28, 2023. Ta at point 1 was consistently lower than at point 2 and point 3. It increased gradually from 08:00 to 10:00, rose sharply after 10:00, peaked at 12:00, and then declined slowly. This pattern is attributed to point 1’s higher tree coverage, experiencing direct G primarily during midday hours. Point 2 and 3, both open spaces with similar spatial layouts, exhibited comparable microclimatic trends. However, point 2 demonstrated lower G due to its relatively higher tree coverage compared to point 3. The distinct microclimatic variations observed between the closely situated points indicate that plant configuration significantly influences the thermal environment within the small-scale pocket park.

#### PET and questionnaire analysis

**Fig. 4:**
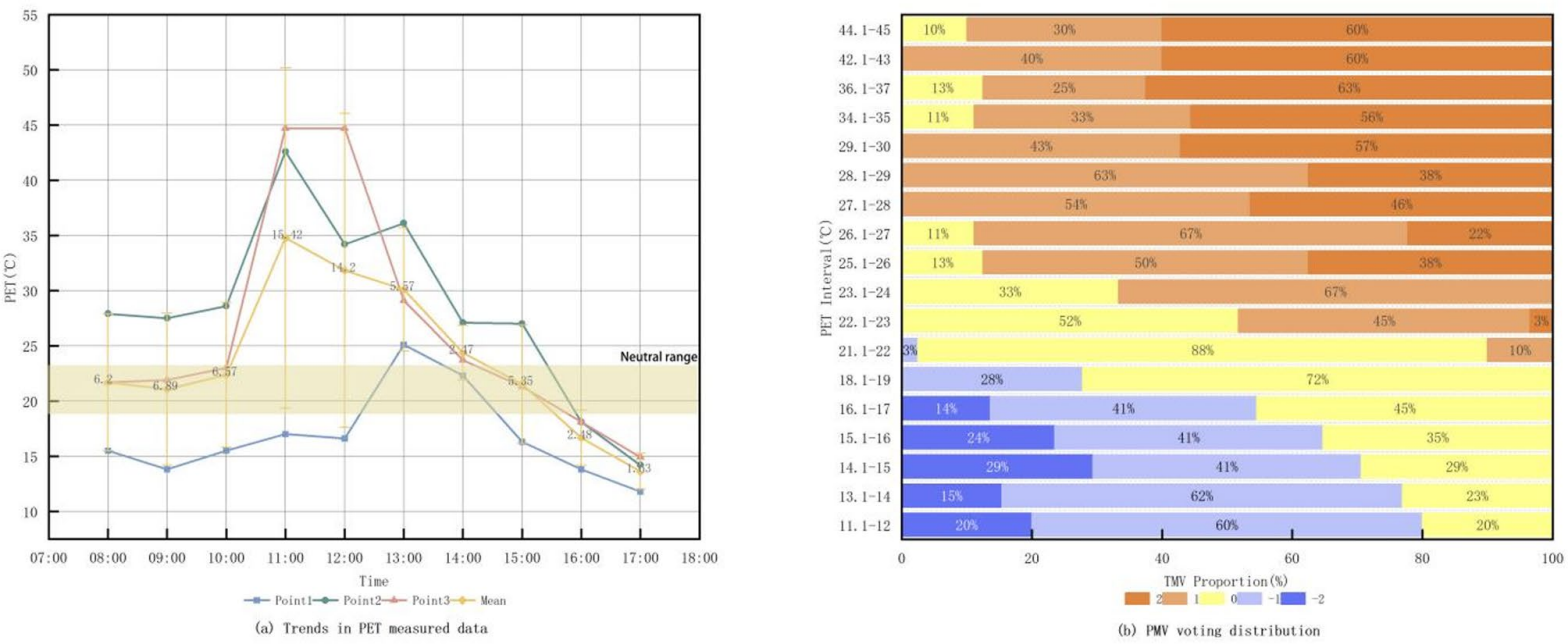
Trends in PET measured data and PMV voting distribution.

Rayman calculated the PET changes of different equal parties in the Freedom Park from 8:00 to 17:00 on September 28, and the TSV vote share in each PET range as shown in Fig. 4. It can be seen that the PET at both measurement point2 and 3 was smoother from 8:00 to 10:00, with a sharp increase followed by a sharp decrease around 11:00, with a larger change. Measurement point 1 had a smoother change in PET because of its higher plant coverage, indicating that the change in its thermal environment was smaller.

Each 1 °C PET was divided into a group, and the TSV voting results of each group were shown in Fig. 4. It can be seen that the TSV results with PET values between 18.1 and 24.0 °C are higher than 50%. This means that the people surveyed in the three sites felt uncomfortable to varying degrees most of the time, and the thermal environment in the site needs to be modified.

### Fitting analysis of PET and MTSV

**Fig. 5:**
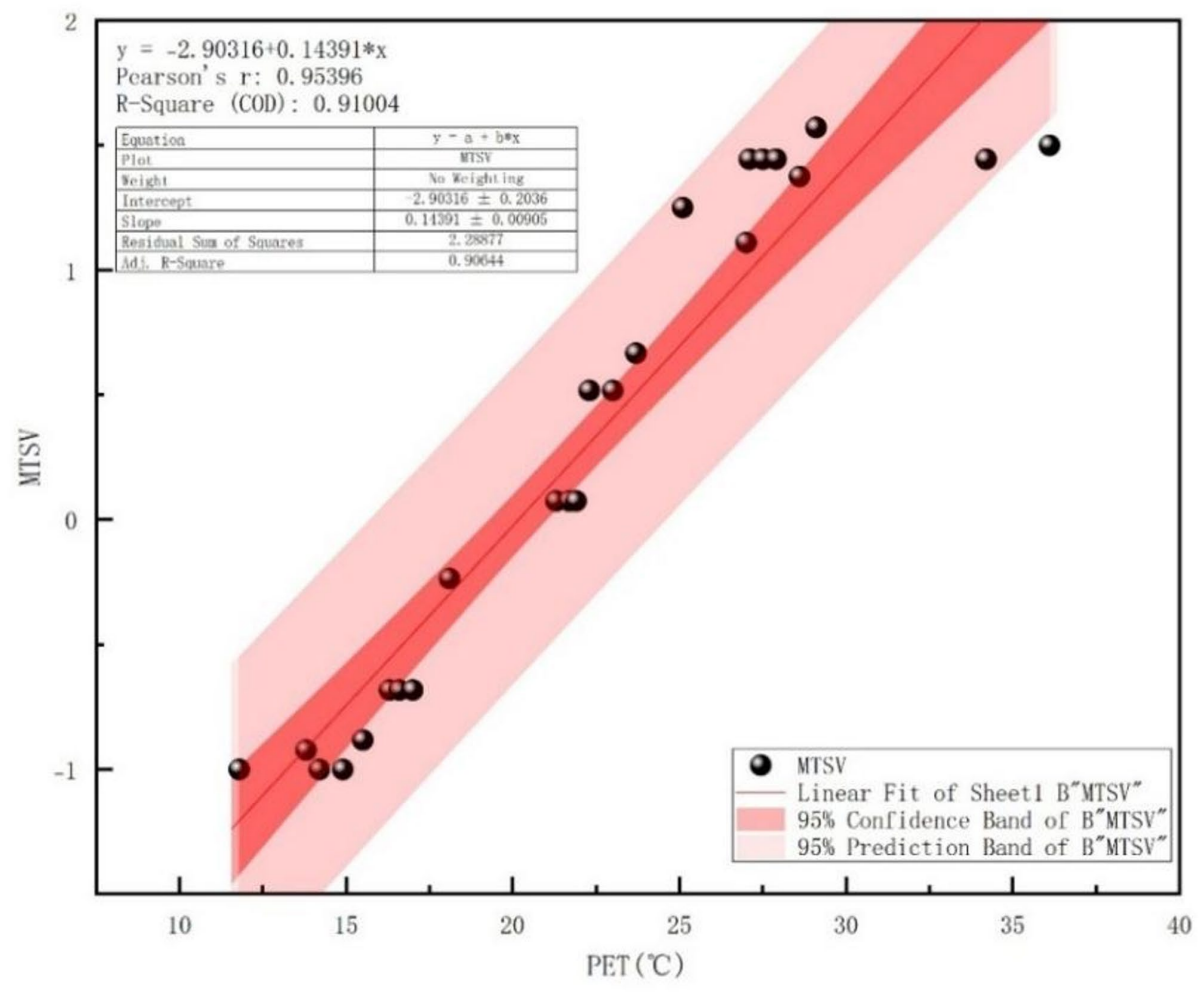
Fitting of mean value of MTSV and PET.

In order to calculate the range of PET thermal comfort range in Changchun Pocket Park more objectively, PETs of every 1 °C were grouped to calculate the MTSV. PET and MTSV were subjected to multivariate linear regression through SPSS and their linear regression relationship was plotted Fig. 5. PET and MTSV and the fitting equations of regression functions were as follows (Eq. 5):

5 $$\:{\text{MTSV}} = 0.14391{\text{PET}} - 2.90316,R^{2} = 0.91,P < 0.001$$

By setting MTSV = 0 in the regression equation, the thermo-neutral temperature for Changchun Pocket Park is calculated as 20.16 °C. The PET comfort range, corresponding to MTSV values of − 0.5 to 0.5 (indicating “comfortable” thermal sensation), is 16.69 °C to 23.63 °C. Similarly, the PET threshold ranges for other thermal sensation categories were calculated and are presented in Table 2. These calculated thermal comfort ranges can inform subsequent pocket park retrofit strategies. The results reveal that, due to regional and seasonal variations, the thermal comfort range identified in this study differ from the internationally recognized European standards. Specifically, they are lower in colder conditions and higher in hotter conditions.

**Table 2:** Transitional season thermal comfort range intervals in Changchun City.

Thermal sensation	MTSV	PET (transitional season in Changchun)(℃)	PET(Common to Europe^38^)(℃)
very cold	−2.5 ~ − 1.5	2.80–9.74	8–13
a little cold	− 1.5 ~ − 0.5	9.74–16.69	13–18
comfortable	− 0.5 ~ 0.5	16.69–23.63	18–23
a little hot	0.5 ~ 1.5	23.63–30.58	23–29
very hot	1.5 ~ 2.5	30.58–37.52	29–35

### Analysis of software simulation results

#### Determination of simulated working conditions

**Fig. 6:**
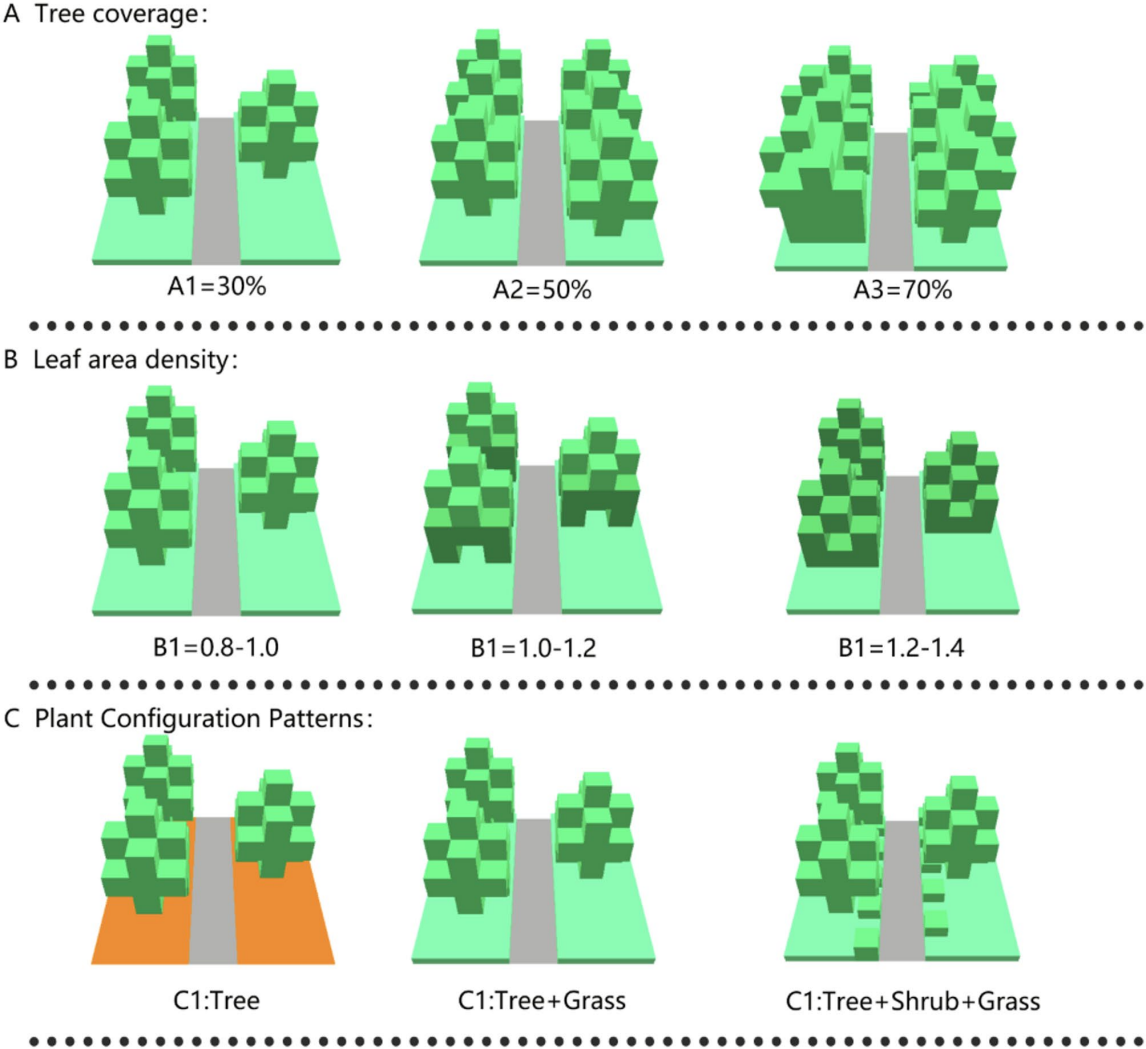
Determination of the model for each working condition (created in ENVI-met Headquarter 5.6.1).

Based on the characteristics of vegetation configuration factors, the simulation established nine working conditions under three factors, as shown in Fig. 6.

1. Tree coverage (A).

Because there are almost no large structures in the functional space of the pocket park under study, the study did not consider the architectural factors. In order to exclude the influence of factors other than plants, this study used tree coverage instead of the sky visibility factor (SVF) as the horizontal variable index of the plant collocation form for the simulation of the working conditions. Tree cover of 30% (A1), 50% (A2), and 70% (A3) were selected as the variables for this condition.

(2) LAD (B).

**Fig. 7:**
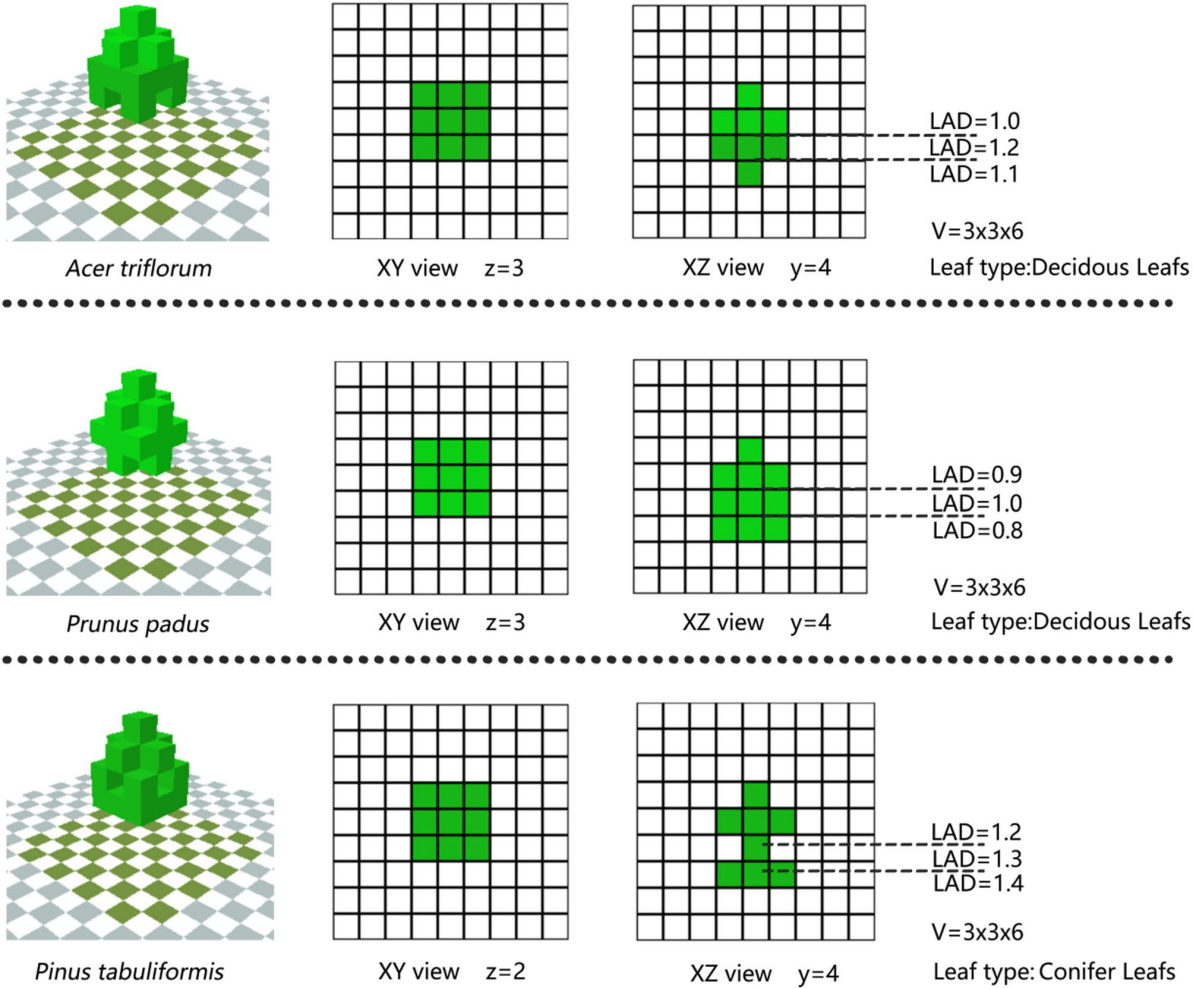
Three-dimensional model of a tree (created in ENVI-met Headquarter 5.6.1).

Because it is difficult to quantify different plant species, the LAD ranges of three common trees in the garden were calculated by LAI to serve as a quantitative index of different plant species. The LAD ranges of 0.8–1.0 (B1), 1.0–1.2 (B2), and 1.2–1.4 (B3) were selected as the variables for this work condition and inputted into the Albreo module of ENVI-met software for plant modeling to differentiate between different species of tree (Fig. 7).

(3) Green patterns (C).

Different vegetation configuration patterns affect the green cover of the site, which in turn affects the thermal environment. When investigating the pocket park site, most of the configurations belonged to the mixed tree-grass pattern. In order to further explore the effect of plant mix form on thermal comfort, the vegetation configuration pattern was selected as a longitudinal variable indicator, and three different patterns, tree (C1), tree-grass (C2), and tree-shrub-grass (C3), were set as the variables for this working condition.

#### Thermal environment simulation results

Through the way of controlling variables, the microclimate simulation data of each level of the three indicators of different levels of plant collocation forms were compared and analyzed respectively. Through analyzing its impact on the microclimate of the pocket park, then judging the mechanism of its impact on the thermal environment of the pocket park.

1. Tree coverage.

The microclimate changes under three different tree cover scenarios, A1 (30%), A2 (50%), and A3 (70%), were comparatively analyzed by means of control variables in the form of the collocation of B1 (LAD = 0.9-1.0) + C1 (trees) as a sample.

**Fig. 8:**
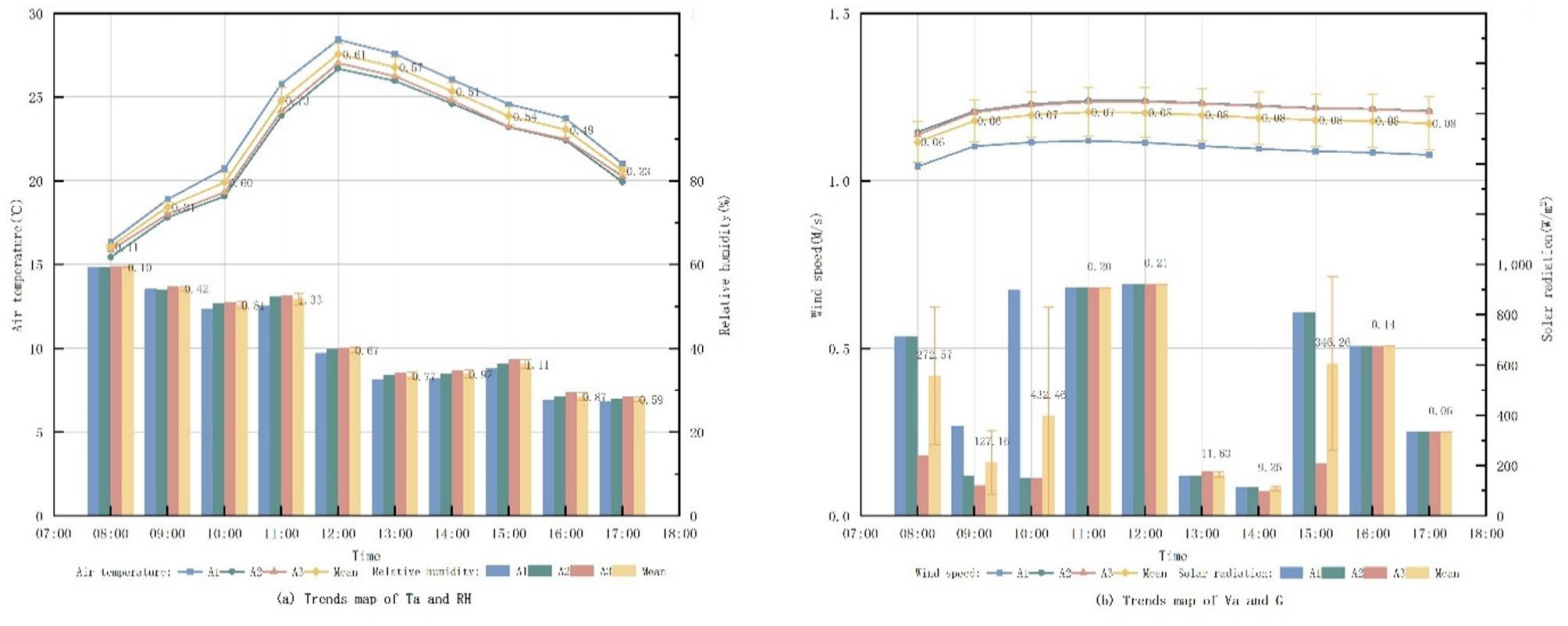
Trends in microclimate factors under different tree coverage.

Figure 8 reveals consistent diurnal trends in Ta across monitoring sites with varying tree coverage: Ta commenced at its lowest point at 08:00, increased steadily, peaked at 12:00 and subsequently declined. Analysis demonstrates that higher tree coverage corresponded with lower overall Ta, indicating a negative correlation. Similarly, RH followed a uniform daily pattern, highest in the morning and decreasing to its minimum by 17:00; increased tree coverage was associated with higher overall RH, showing a positive correlation. Va exhibited a consistent daily trend; however, Va at site A1 was significantly lower than at A2 and A3, while Va levels between A2 and A3 were comparable with only a marginal difference, suggesting no clear correlation with tree coverage. G showed a pattern similar to Ta, rising sharply after 10:00, peaking at 12:00, and then decreasing rapidly at the roadside measurement point under direct noon sun. Before 10:00, under shade from the adjacent eastern plant community, G decreased with increasing canopy coverage, indicating a negative correlation under shaded conditions.

(2) Tree species.

Using LAD to quantify plant species, the microclimate changes in the case of different tree species in B1 (LAD = 0.8–1.0), B2 (LAD = 1.0–1.2), and B3 (LAD = 1.2–1.4) were comparatively analyzed by means of controlling variables in the form of the pairing of A3 + C1 as a sample.

**Fig. 9:**
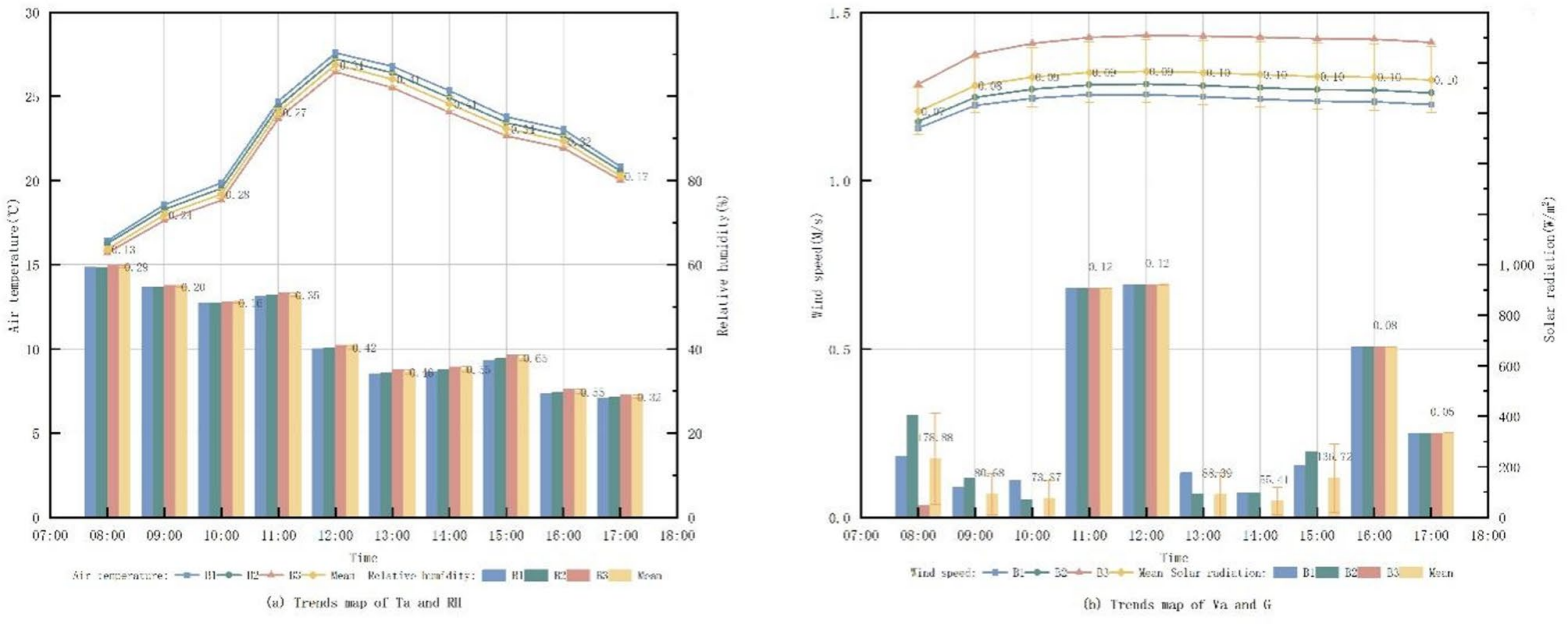
Trends in microclimate factors under different LAD.

From the Fig. 9, the daily trend of Ta at the test site was the same throughout the day for different tree species. By comparing the Ta of the three different LAD, it can be seen that as the LAD value rises, the Ta as a whole begins to decrease, indicating that the LAD is negatively correlated with the Ta. The RH with the highest humidity in the morning, decreasing with time, and reaching a minimum at 17:00. By comparing the RH at different LAD, it can be seen that the overall trend is not much different, and when the LAD rises, the RH as a whole starts to rise as well, indicating that the LAD is weakly and positively correlated with the RH. The trend of Va is the same throughout the day. By comparing the Va s at different LADs, it can be seen that as the LAD rises, the Va at the measurement point also rises, indicating a positive correlation between LAD and Va. The trend of G is similar, it starts to rise sharply after 10:00 and reaches the maximum value at 12:00, and then starts to fall sharply. The reason is that the measurement point is located in the area where trees are planted on the east and west sides of the road, and there are no trees shading the location of the measurement point when the sun is shining directly at noon. By comparing the trend of G at the location of the measurement point before 10:00 and at 13:00, when the measurement point is shaded by trees, it can be seen that as LAD rises, G decreases, which indicates that LAD is negatively correlated with G.

(3) Green patterns.

The microclimate changes under three different green patterns of C1 (tree), C2 (tree + grass), and C3 (tree + shrub + grass) were comparatively analyzed by means of controlling variables, using the collocation form of A1 (30%) + B1 (LAD = 0.8–1.0) as a sample.

**Fig. 10:**
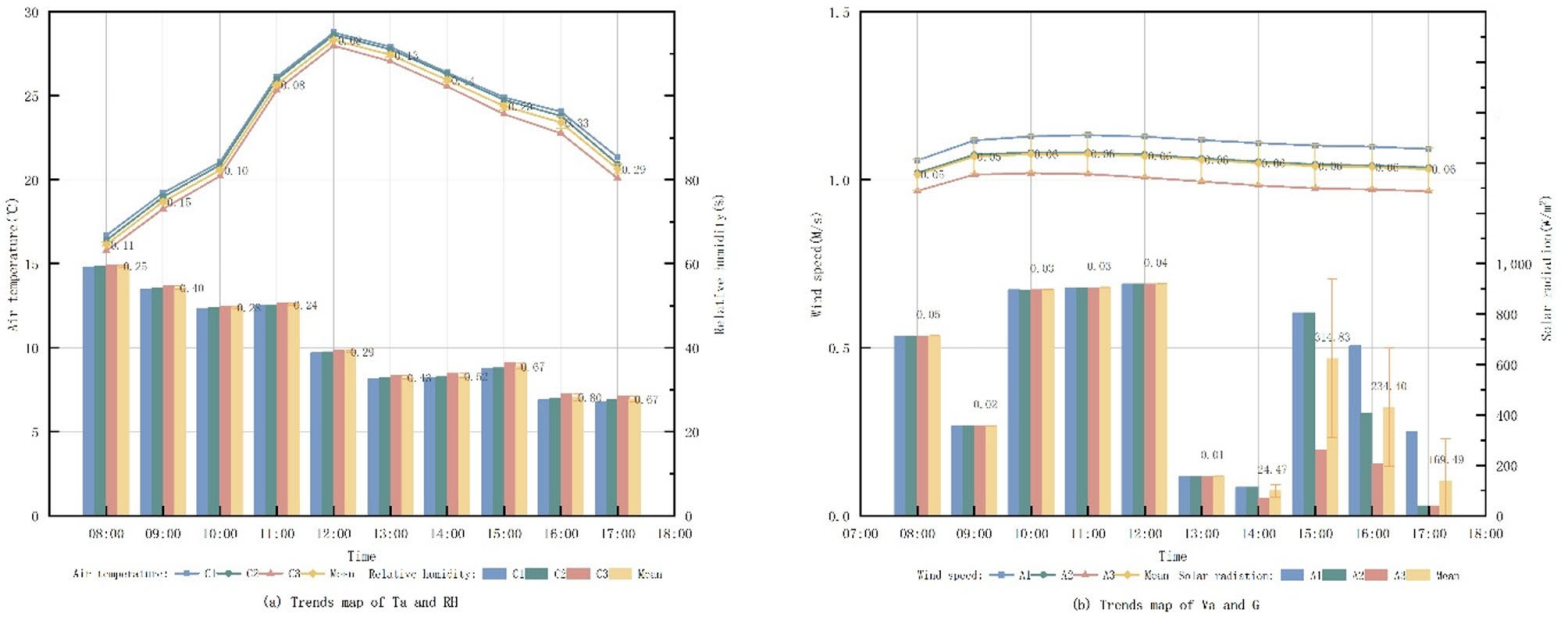
Trends in microclimate factors under different vegetation configuration patterns.

From the Fig. 10, it can be seen that the daily trend of Ta at the testing site is the same in different green patterns modes. By comparing the Ta of the three impassable vegetation configuration modes, it can be seen that the difference between C1 (tree) and C2 (tree + grass) is very small, and the Ta of C3 (tree + shrub + grass) after 12:00 decreases more than that of the other two conditions, which indicates that the planting of shrubs has a greater impact on the Ta. The RH have the same trend. By comparing the RH of the three impassable vegetation configuration modes, it can be seen that the difference between C1 (tree) and C2 (tree + grass) is also very small, and the RH of C3 (tree + shrub + grass) at 13:00 rises more than that of the other two conditions, which indicates that the planting of shrubs has a greater impact on the RH. The trend of Va is the same. By comparing the Va of the three different green patterns, it can be seen that the Va gradually becomes weaker with the richness of the vegetation configuration patterns, indicating that the richness of the green patterns and the Va have an obvious negative correlation. The trend of G is similar, and there is an obvious difference after 13:00. By comparing the G after 13:00 of the three different green patterns, it can be seen that with the abundance of green patterns, the G gradually becomes weaker, indicating that the abundance of green patterns is negatively correlated with the G.

#### Degree of influence of different vegetation configuration on thermal comfort

Because of the complexity of the way in which different plant factors affect PET, it is not possible to analyze the influence mechanism by simple comparison, so the orthogonal test method is used to evaluate the degree of influence of each element on thermal comfort.

To determine the influence of each element on thermal comfort by exploring the tree cover, LAD, and vegetation configuration mode, the orthogonal test factor level table was established by taking PET, an outdoor thermal environment evaluation index, as the test index, and considering three elements, namely, tree coverage (A), LAD (B), and green patterns (C), in combination with the actual element matching and the simulated environment, as shown in Table 3.

**Table 3:** The classification of each factor grade in orthogonal experiment considering the effects of the interaction between the elements on thermal comfort, the test program for the functional space was selected using the table below. The test was calculated only for the nine working conditions in the table due to the consideration of the interaction between the elements, as shown in Table 4.

Factor level	Tree coverage (A)	LAD (B)	Green patterns (C)
1	30%	0.8–1.0	Tree
2	50%	1.0–1.2	Tree + grass
3	70%	1.2–1.4	Tree + shrub + grass

**Table 4:** Orthogonal experimental model design.

Name	A	B	C	D	PET
1	1	1	1	1	31.06
2	1	2	3	2	27.24
3	1	3	2	3	25.32
4	2	1	3	3	24.93
5	2	2	2	1	25.18
6	2	3	1	2	24.57
7	3	1	2	2	24.14
8	3	2	1	3	25.14
9	3	3	3	1	21.24

**Fig. 11:**
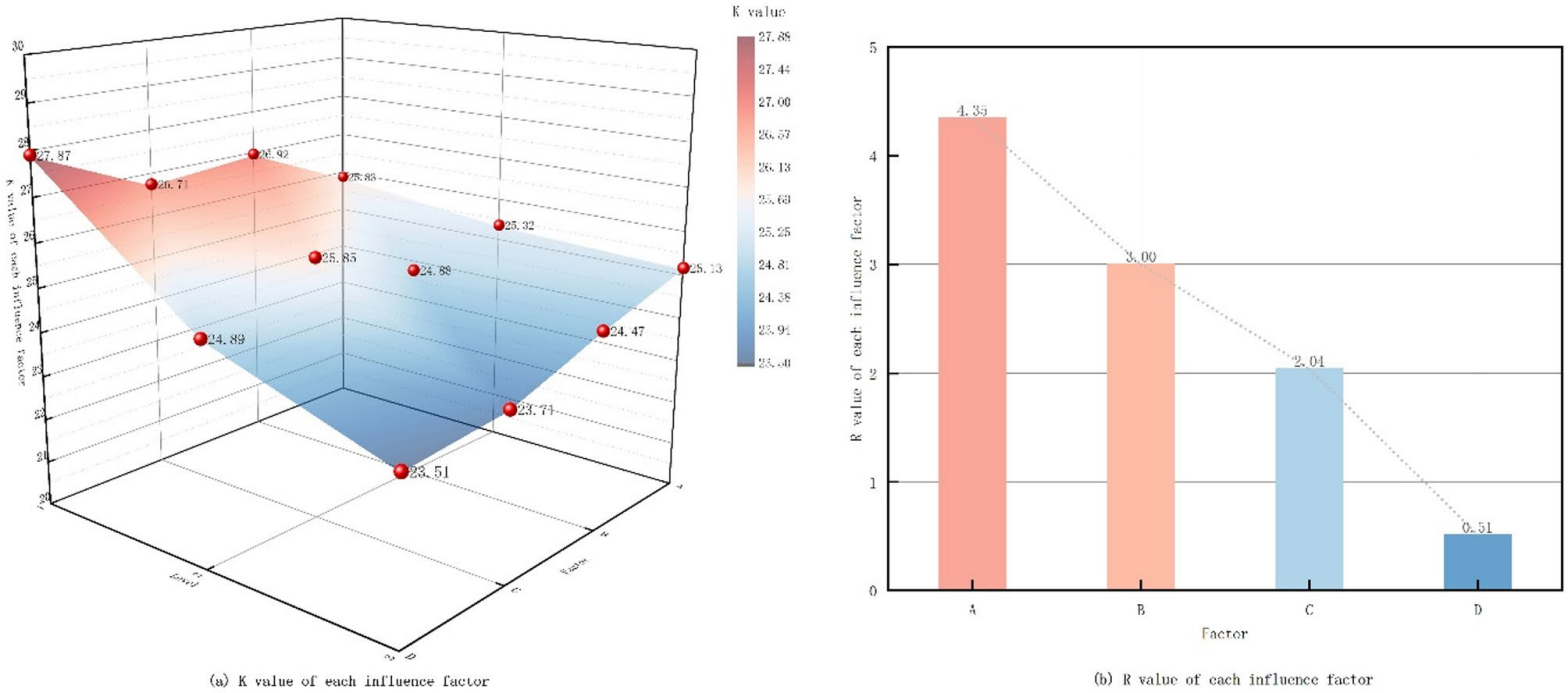
K value of each influence factor (analyzed with IBM SPSS Statistics 27; plotted using Origin Pro 2025).

For the Fig. 11, by comparing the level indicators of each factor and K1, K2 and K3, the level ordering between the factors was analyzed: according to the results of the orthogonal test, it was seen that the larger the value of the extreme difference (R), the higher the degree of influence. It can be seen that the tree coverage (A) had the greatest influence on the PET of the experimental model, with the extreme difference mean at 4.34, the LAD had the second largest influence, with a value of 3.00, and the green patterns had the smallest influence, about 2.04.

For the Table 5, by using SPSS to analyze the ANOVA of each influence factor in the model, the significance is less than 0.05 means that the factor has a significant effect on the test indicator, and greater than 0.05 means that the effect is not significant. According to the table, the effect of arbor coverage (A) in the model is less than 0.05 and has a significant effect on PET. Therefore, the significance of influencing the thermal comfort of the freedom park is in the following order: tree coverage > LAD > green patterns.

**Table 5:** ANOVA variance analysis of each influence factor.

Root	Type III sum of squares	Degrees of freedom	Mean square	F	Significance
Correction model	54.561^a^	6	9.093	23.308	0.042
Intercept	5817.621	1	5817.621	14,911.455	0
A	29.871	2	14.936	38.282	0.025
B	14.328	2	7.164	18.362	0.052
C	10.362	2	5.181	13.28	0.07
Inaccuracies	0.78	2	0.39		
Total	5872.963	9			
Amended total	55.341	8			

^a^R^2^ = 0.986( Adjusted R^2^ = 0.944)

#### Optimized thermal comfort validation

**Fig. 12:**
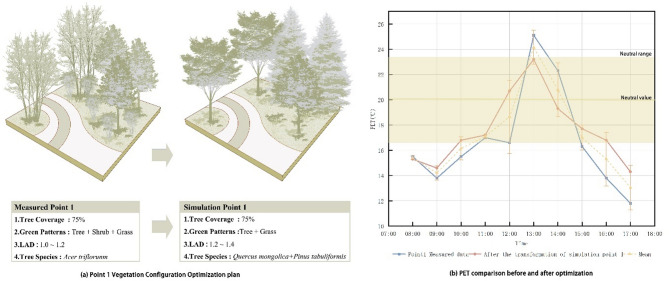
Point 1 vegetation configuration optimization plan and PET comparison before and after optimization. (**a**) was created in Adobe Photoshop 2023; (**b**) was plotted using Origin Pro 2025.

Based on the orthogonal experiment results, we optimized the design for Point 1 (Fig. 12). We retained 70% of the original site’s tree coverage. The original mixed planting pattern of tree、shrub and grass was replaced with a tree and grass combination. The primary tree species *Acer triflorum* (LAD = 1.0) was substituted with *Pinus tabuliformis* (LAD = 1.2) and *Quercus mongolica* (LAD = 1.4). The revised vegetation configuration model for Point 1 was then imported into the ENVI-met software for simulation.

The landscape optimization implemented at Point 1 has resulted in a significant and consistent improvement in the microclimate, as simulated by ENVI-met. The key outcome is a reduction in PET, particularly during the peak heat hours (reduction of ~ 4 °C), shifting conditions closer to or within the thermal comfort range for a larger portion of the day. This demonstrates the effectiveness of the design changes in mitigating heat stress.

## Discussion

### Variations in thermal comfort range

Because different climatic zones lead to different variations in the thermal environment within different geographical settings, this results in different criteria for evaluating thermal comfort for people within different geographical environments. In the same environment, the evaluation of the comfort level of the thermal environment varies due to individual differences in the population. This study utilized the MTSV obtained from the field study with the PET, regression linear operations were performed by SPSS software, a PET comfort range of 16.69–23.63 °C was obtained for the transition season in the cold urban pocket park. When PET was 20.37 °C, the population of the area considered the thermal environment to be the most comfortable. Sihan Xue et al. calculated thermoneutral PET thresholds for the transition season in urban parks in cold regions of Zhengzhou City is 4.9–24.3 °C, comparison with it shows a wider range of thermal neutrality thresholds at lower latitudes in Zhengzhou compared to Changchun^39^. High humidity elevates PET via suppressed heat dissipation; low wind reduces cooling; intense radiation increases heat sensation/PET. These prevalent low-latitude conditions shape thermal comfort range and drive local adaptation. The wider range of thermal comfort is a result of the wider range of thermal acceptability of the local population. Cheng Wang calculated thermal neutrality thresholds of 19.2–28.7 °C for PET in the transition season in the hot and humid Lingnan region^40^, higher than the thermal neutrality threshold of the Changchun, It indicates that the heat tolerance of the population in the southern region is higher than that of the population in the cold northern region, while citizens in the cold region tend to favor cooler thermal environments during the transition season.

### Impact of vegetation configuration on microclimate

Regulating the thermal environment by optimizing the vegetation configuration in pocket parks is an effective way to enhance the thermal comfort in pocket parks. Wiebke Klemm considered the effect of tree coverage and green patterns form on the thermal environment^41^ and did not consider the differences caused by different tree species. Zhao et al. examined the mechanisms by which various morphological characteristics of individuals of different tree species affect thermal comfort^12^ and did not consider the effects of other vegetation configuration factors. Perry J. Hardin et al. combined LAI with plant hierarchies with the intention of ameliorating the urban heat island effect by optimizing vegetation configurations^42^. Mohamad Fahmy et al. explored how trees affect the thermal environment using LAI and LAD as the main indicators^43^. In this study, it can be seen that different indicators of each level of vegetation configuration factors affect different microclimate factors in different ways. By increasing the tree coverage can effectively reduce the temperature and G, but it also causes the RH to rise. In the study area Va also rises when the tree coverage is increased, this is because the trees on both sides of the road will form a ventilation corridor, resulting in increased Va at the measurement point location. This is because the trees on both sides of the road create ventilation corridors, which result in higher Va at the location of the measurement points. However, when the tree coverage continues to increase to a certain level and encloses the site too much, the ventilation corridors are destroyed and the Va are reduced.

### Impact of vegetation configuration on thermal comfort

Analysis of vegetation’s microclimate influence reveals non-linear relationships, necessitating holistic evaluation of plant configurations on PET. Orthogonal testing identified vegetation structure (tree coverage) as the most critical factor for improving thermal comfort in pocket parks, followed by LAD and planting patterns. Optimization should prioritize increasing tree coverage, then adjusting LAD through species selection, and finally refining planting diversity. Enhanced LAD reduces temperature and G while elevating humidity and facilitating ventilation corridors, albeit potentially increasing Va. Enriched vegetation configurations (e.g., adding shrubs) further mitigate temperature and boost humidity, though excessive shrub use may impede airflow.

Thus, vegetation is pivotal to pocket park thermal environments. Design strategies must integrate site-specific needs with targeted adjustments to vegetation structure, LAD, and planting patterns, balancing their interdependent effects on microclimate parameters for optimal comfort outcomes.

### Design strategy

Through ENVI-met’s simulation of all working conditions of different levels of vegetation configuration and indicators in the study site, we obtained the all-day average PET value of each working condition, and then brought it into the comfortable range of PET in cold cities to obtain the optimization strategy of plant mixing form for the study site. The average PET of the whole day in the environment can reach the standard of “comfortable” only when the percentage of tree coverage > 50%, the LAD > 1.2–1.4 and the vegetation configuration pattern is more abundant than tree + grass. Since the tree coverage has the highest influence among the plant mixing forms, in the subsequent optimization process, under other conditions, the tree coverage should be increased to more than 50%, and the tree species with LAD greater than 1.2 should be selected for planting. This can effectively reduce the Ta and G, and increase the RH in the site.

**Fig. 13:**
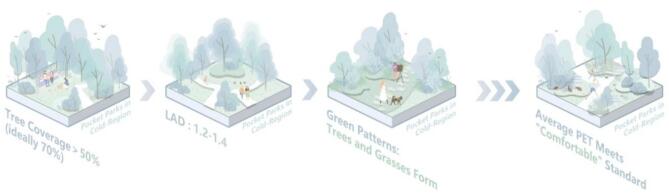
Design strategy of vegetation configuration (created in Adobe Photoshop 2023).

### Impacts and limitations

While this study advances thermal comfort optimization in cold-region pocket parks, four limitations warrant attention: (1) Field measurements prioritized vegetation configuration over synergistic factors (building morphology, surface materials, anthropogenic heat); (2) Leaf area index estimations relied on literature data rather than site-specific measurements; (3) Spatial resolution constraints from limited monitoring points may underrepresent microclimatic heterogeneity; (4) Exclusive focus on transitional seasons precludes seasonal comparative analysis. Future research should integrate multi-source sensing networks with high-resolution simulations to refine parameter accuracy, while extending investigations to summer/winter periods to capture seasonal dynamics. We advocate for developing interdisciplinary frameworks combining micrometeorological monitoring, parametric modeling, and machine learning to enhance the climate resilience of micro-scale green spaces. Thermal comfort improvement goals should also be considered in conjunction with long-term maintenance feasibility, affordability, and ecological sustainability.

## Conclusion

This integrated field-simulation study elucidates vegetation configuration impacts on transitional season thermal comfort in cold-region pocket parks through three principal findings. First, parametric analysis revealed tree coverage as the dominant regulator, exhibiting negative correlations with Ta and G, while LAD modulated humidity-wind balance. Second, psychophysiological modeling established 16.69–23.63 °C as the PET comfort range, anchored by a thermal neutral temperature of 20.16 °C. Third, orthogonal experimental design quantified the hierarchical significance: tree coverage > LAD > green patterns. These findings yield evidence-based design protocols: (1) tree coverage should exceed 50%, preferably reaching 70%; (2) select species maintaining LAD 1.2–1.4; (3) optimal green patterns should combine tree and grass elements. Through simulations of the optimized plant configuration plan for Freedom Park, we validated the effectiveness of this strategy in enhancing thermal comfort within cold-region pocket parks. This approach demonstrates practical application value for improving thermal conditions in similar sites. The study advances microclimate-sensitive design paradigms for cold urban ecosystems.

## Data Availability

The datasets generated and analyzed during the current study are not publicly available due to but are available from the corresponding author on reasonable request.

## Declarations

### Author statement

The work described has not been published previously. The article is not under consideration for publication elsewhere. The article’s publication is approved by all authors and tacitly or explicitly by the responsible authorities where the work was carried out.
